# Ribosomal RACK1 Regulates the Dendritic Arborization by Repressing FMRP Activity

**DOI:** 10.3390/ijms231911857

**Published:** 2022-10-06

**Authors:** Nicla Romano, Bruna Di Giacomo, Veronica Nobile, Antonella Borreca, Daniela Willems, Francesca Tilesi, Elisabetta Catalani, Manasi Agrawal, Kristy Welshhans, Sara Ricciardi, Davide Cervia, Marcello Ceci

**Affiliations:** 1 Department of Ecological and Biological Sciences (DEB), University of Tuscia, 01100 Viterbo, Italy; 2 Max Planck Institute for Biology of Ageing, 50931 Cologne, Germany; 3 Institute of Genomic Medicine, Fondazione Policlinico Universitario, “A. Gemelli” IRCCS, 00168 Rome, Italy; 4 Institute of Neuroscience, CNR-National Research Council, Vedano al Lambro, 20133 Milan, Italy; 5 Humanitas Clinical and Research Center-IRCCS, Rozzano, 20089 Milan, Italy; 6 Department for Innovation in Biological, Agro-food and Forest systems (DIBAF), University of Tuscia, 01100 Viterbo, Italy; 7 School of Biomedical Sciences, Kent State University, Kent, OH 44243, USA; 8 Brain Health Research Institute, Kent State University, Kent, OH 44243, USA; 9 Department of Biological Sciences, University of South Carolina, Columbia, SC 29208, USA; 10 Istituto Nazionale Genetica Molecolare “Romeo ed Enrica Invernizzi”, 20122 Milan, Italy; 11 Bioscience Department, Università degli Studi di Milano, 20133 Milan, Italy

**Keywords:** neuron differentiation, RACK1, FMRP, translation, ribosomes, neurons, neuroblastoma

## Abstract

FMRP is an RNA-binding protein that represses the translation of specific mRNAs. In neurons, its depletion determines the exaggerated translation of mRNAs leading to dendritic and axonal aberrant development, two peculiar features of Fragile X syndrome patients. However, how FMRP binds to translational machinery to regulate the translation of its mRNA targets is not yet fully understood. Here, we show that FMRP localizes on translational machinery by interacting with the ribosomal binding protein, Receptor for Activated C Kinase 1 (RACK1). The binding of FMRP to RACK1 removes the translational repressive activity of FMRP and promotes the translation of *PSD-95* mRNA, one specific target of FMRP. This binding also results in a reduction in the level of FMRP phosphorylation. We also find that the morphological abnormalities induced by *Fmr1* siRNA in cortical neurons are rescued by the overexpression of a mutant form of RACK1 that cannot bind ribosomes. Thus, these results provide a new mechanism underlying FMRP activity that contributes to altered development in FXS. Moreover, these data confirm the role of ribosomal RACK1 as a ribosomal scaffold for RNA binding proteins.

## 1. Introduction

Until some years ago, the ribosome was considered to be a homogenous structure, essential for the translation of mRNAs. However, numerous studies have recently found that the protein composition of ribosomes is heterogeneous among mammalian tissues [1] and that a variety of ribosomal proteins, outside of those directly constituting the ribosomes, participate in the translation of specific mRNAs and/or are an integral part of pathways stimulating global translation [2,3]. Among these ribosomal proteins, the Receptor for Active C Kinase 1 (RACK1) is emerging as a key ribosomal player. RACK1 was initially isolated as a scaffold protein for active PKCβII [4]. Additional studies found that RACK1 interacts with numerous proteins, such as Src, JNK, ERK and FAK kinases [5,6,7], cyclic-AMP specific phosphodiesterase isoform D5 (PDE4D5) [8], Integrin β1 [9] and many others. Moreover, as RACK1 interacts with a large variety of proteins, it is involved in many cellular processes, ranging from cell cycle regulation to cell adhesion [10,11,12,13].

RACK1 is also a ribosomal binding protein [14]. Biochemical experiments have demonstrated that the binding of RACK1 to ribosomes is so strong that RACK1 should be considered a ribosomal protein [15]. Crystallographic studies have shown that RACK1 is located at the back of the head of the 40S subunit, proximal to the exit channel of the mRNA [16]. However, despite these findings, the function of ribosomal RACK1 is not yet fully defined. One role of RACK1 is as a ribosomal scaffolding protein for kinases, thus providing a hub integrating cell signalling and global protein synthesis. Indeed, PKC by interacting with RACK1 on ribosomes, phosphorylates eIF6, thereby removing its repressor translational activity and stimulating global protein synthesis [17]. In addition, the PKCβII-RACK1 complex modulates the recruitment and the phosphorylation of eIF4E and eIF4G on ribosomes [18,19,20,21]. The binding of RACK1 with another kinase, JNK, monitors the quality of newly synthesized polypeptides (NSP) [22]. In addition, ribosomal RACK1 can also modulate the recruitment of specific RNA-binding proteins (RBPs) on translational machinery. Indeed, the binding of ribosomal RACK1 with ZBP1 promotes the translation of β-actin mRNA, mediating the phosphorylation of ZBP1 by Src kinase [23]. RACK1 also mediates the binding between translational machinery and TDP-43, which is an RBP involved in Amyotrophic Lateral Sclerosis (ALS). Interestingly, the depletion of RACK1 on ribosomes reduces the formation of TDP-43 cytoplasmic inclusions, which are distinct features of ALS disease [24]. Collectively, these findings suggest that ribosomal RACK1 may contribute to the translation of specific mRNAs. In line with this suggestion, it has been shown that ribosomal RACK1 regulates the cell cycle of neuroblastoma and hepatocarcinoma cells, by modulating the translation of the cell cycle and survival genes [18,25].

Although the involvement of RACK1 in many cellular processes is now well established, its role in differentiated cells is still unclear [26]. In primary neuron cultures, RACK1 regulates axon growth by modulating the formation of neuronal-specific adhesions, termed point contacts [12,27]. As previously reported, the interaction of RACK1 with ZBP1 leads to the translation of β-actin mRNA, which is essential for neurite outgrowth [23]. These findings suggest that RACK1 may participate in the local translation of specific mRNAs by binding to other RBPs to promote neuronal development. Another RBP involved in dendritic and axonal development is the Fragile X mental retardation protein (FMRP). FMRP is localized to dendrites and synapses where it regulates mRNA transport and local protein synthesis of specific mRNAs necessary for neuronal development and synaptic plasticity [28]. Loss of FMRP results in Fragile X syndrome (FXS), the most common monogenetic form of inherited intellectual disability and autism [29]. Patients with FXS display dendritic spine defects and neurodevelopmental delay [30]. Fmr1 knockout mice, a mouse model of FXS, show a loss of translational repression, resulting in excessive protein synthesis due to increased translation of FMRP mRNA targets [31].

Here, we show that ribosomal RACK1 controls the dendritic arborization development and the dendritic spine density of neurons cultured in vitro. This function interferes with the repressor translational activity of FMRP protein, as demonstrated by the rescue of dendritic spine defect in Fmr1 siRNA neurons induced by the up-regulation of the ribosomal mutated RACK1. To understand this pathway, we find that ribosomal RACK1 recruits FMRP on ribosomes/polyribosomes to modulate the translation of the FMRP mRNA target, PSD-95 mRNA. Thus, these results provide a new mechanism underlying FMRP activity and confirm the role of ribosomal RACK1 as a ribosomal scaffold for RBPs.

## 2. Results

### 2.1. Ribosomal RACK1 Is Required for the Development of Dendritic Arborization and the Dendritic Spines

**Figure 1:**
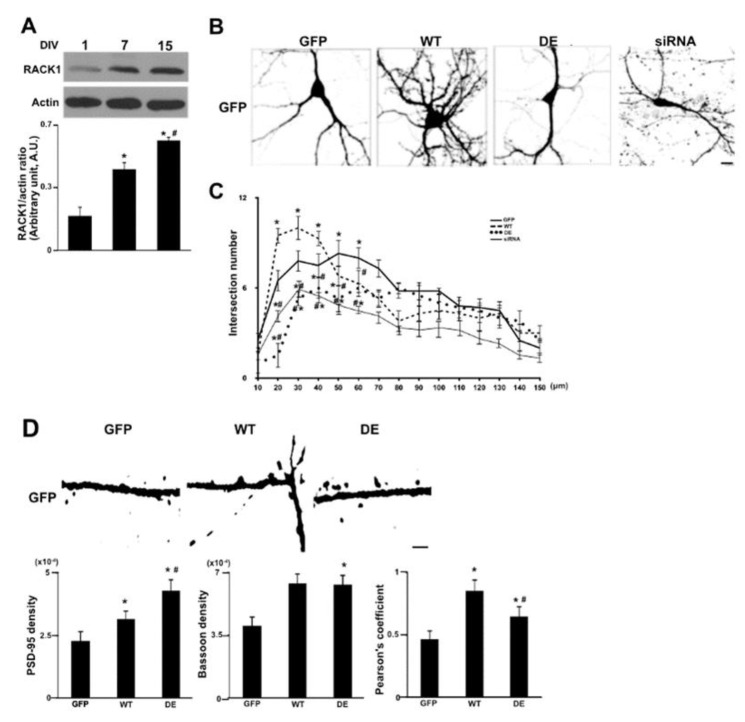
The ribosomal RACK1 regulates dendritic arborization. (**A**) Immunoblotting for RACK1 and actin on total protein lysate from embryonic mouse hippocampal neurons cultured from 1 DIV to 17 DIV. (**Lower**) The graph reports the means of three independent experiments of density ratio between RACK1 and actin bands (mean ± S.D.). Student’s T-test was used to calculate *p* values: * *p* ≤ 0.05 vs. 1 DIV; # *p* ≤ 0.01 vs. 7 DIV. B) Immunofluorescence for GFP on hippocampal neurons transfected at 15 DIV with GFP, GFP/RACK1_WT-his-myc_ (WT in the figure), GFP/RACK1_DE-his-myc_ (DE) or RACK1 siRNA (siRNA). Scale 10μm. (**C**) The graph of Scholl analysis performed on cells in (**B**). The values were measured as means of the number of neurite intersections measured by Sholl analysis. Data are graphed as mean ± S.D. Student’s T-test was used to calculate *p* values: * *p* ≤ 0.01, vs. GFP; # *p* ≤ 0.05 vs. RWT_-his-myc;_ (**D**) Graphic of PSD-95, Bassoon density and Pearson’s coefficient of puncta between PSD-95 and Bassoon measured by immunofluorescence experiments. Graph reporting the correlation measured by Pearson coefficient of the puncta signal between PSD-95 and Bassoon in Supplemental Figure S2. All bars represent mean ± SD; n.70 puncta from three independent experiments. One-way ANOVA with Tukey’s Post Hoc test. Data are graphed as mean ± S.E.* *p* ≤ 0.005, vs. GFP; # *p* ≤ 0.05, vs. WT.

To understand the role of RACK1 in neuronal development we initially investigated its expression in embryonic mouse hippocampal neurons cultured from 1 day in vitro (DIV) to 15 DIV. Using RACK1 immunoblotting we found that the RACK1 expression increased from 1 to 15 DIV, being significant already at 7 DIV (Figure 1A). This suggested a correlation between RACK1 up-regulation and progressive neuronal maturation. To next discriminate which ribosomal forms of RACK1 between the no associated and associated ones are involved in neuronal differentiation, we compared the morphology of 15 DIV mouse hippocampal neurons expressing a mutant form of RACK1 that has a low ribosomal affinity (R36D/K38E, RACK1_DE_) with that of neurons expressing wild-type RACK1 [19,24], both fused to a histidine-myc tag (RACK1_DE-his-myc_ and RACK1_WT-his-myc_). To visualize the complete neuronal shape, the RACK1_DE-his-myc_ and RACK1_WT-his-myc_ were co-transfected with a green fluorescence protein (GFP) that diffuses in all neuronal compartments. The confocal analysis showed an increase in dendritic arborization in neurons overexpressing RACK1_WT-his-myc_ compared to those of controls (neurons expressing GFP only). In contrast, the dendritic arborization was decreased in neurons overexpressing RACK1_DE-his-myc_ (Figure 1B). In addition, we also compared the morphology of neurons downregulating RACK1 by siRNA to all other neurons and we found that the RACK1 downregulation determined a decrease of dendritic arborization likely to observed in RACK1_DE-his-myc_ neurons (Figure 1B). To corroborate this observation, we also measured the number of dendritic intersections by Sholl analysis. Consistent with previous results, we observed an increase in the number of intersections in RACK1_WT-his-myc_ neurons within a radial distance between 20 μm and 50 μm, and a decrease in the number of the intersections in this same radial distance in RACK1_DE-his-myc_ and RACK1 siRNA neurons (Figure 1C). These data clearly indicate that the RACK1 mutant affects dendritic development and suggest that the ribosomal RACK1 is the main form involved in neuronal differentiation.

Next, as along dendrites specific structures called dendritic spines are reached by many terminal axons to form excitatory synapses [32], we wondered whether the dendritic spines density, defined as the number of Post-Synaptic Density 95 (PSD-95) puncta per dendritic length measured by PSD-95 immunofluorescence, could also undergo any alteration by the RACK1 up-regulation. RACK1_WT-his-myc_ expression led to an increase in PSD-95 density as compared to controls. However, in contrast to our results on dendritic arborization, the neurons expressing RACK1_DE-his-myc_ showed more PSD_95 density than any other group (Figure 1D and bar graph).

In line with these results, we also found that SH-SYS5 human neuroblastoma cells transfected with RACK1_DE-his-myc_ and RACK1_WT-his-myc_ expressed more PSD-95 protein compared to controls, indicating that the increase of PSD-95 density corresponded to an increase of its protein level (Supplemental Figure S1A). Moreover, as observed in neurons, the expression of PSD-95 protein was more elevated in cells expressing that RACK1 mutant than in those expressing the RACK1 wild-type form. As we previously reported that ribosomal RACK1 regulates the translation of mRNAs encoding cell cycle genes in neuroblastoma cells [25] and β-actin mRNA in neuronal growth cones [23], we wondered if the increase in PSD-95 expression occurred at the translational level. To verify that this is indeed the case, we performed a qRT-PCR assay to measure the PSD-95 mRNA level in RACK1_DE-his-myc_, RACK1_WT-his-myc_, and control neuroblastoma cells. It was found that the amount of PSD-95 mRNA, normalized with respect to the 18S rRNA level, reduced cells up-regulating both forms of RACK1 as compared to controls (Supplemental Figure S1B). These data clearly support the hypothesis that the expression of PSD-95 is indeed controlled by ribosomal RACK1 at the translational level. Altogether our results also indicate that ribosomal RACK1 is likely to be involved in the dendritic spine development and the translational control of PSD-95 mRNA.

Next, we valued whether the increase of dendritic spine density induced by the RACK1 mutant in the neurons could form synapses. For this, we counted the number of puncta where PSD-95 and the pre-synaptic marker Bassoon colocalized per dendritic length and reported as Pearson’s coefficient. In neurons expressing RACK1_DE-his-myc_ or RACK1_WT-his-myc_, the number of colocalized puncta was higher than controls, probably also due to an increase in Bassoon density, as seen for PSD-95 (Figure 1D and Supplemental Figure S2A). Furthermore, comparing RACK1_DE-his-myc_ neurons with that of RACK1_WT-his-myc_, the colocalization of PSD-95 was higher in neurons expressing RACK1_DE-his-myc_ than in the RACK1 mutant, although both neurons showed similar levels of Bassoon density (Figure 1D). These results revealed that although RACK1 mutant expression induced an elevated number of the dendritic spines, only a part of them formed the synapses. Instead, most of the dendritic spines induced by RACK1_WT-his-myc_ overexpression were reached by the pre-synaptic terminal. Thus, these data further support the role of ribosomal RACK1 in the development of dendritic spines and synaptic formation.

### 2.2. Ribosomal RACK1 can Rescue Morphological Neuronal Defects Caused by Fmr1 Knockdown

**Figure 2:**
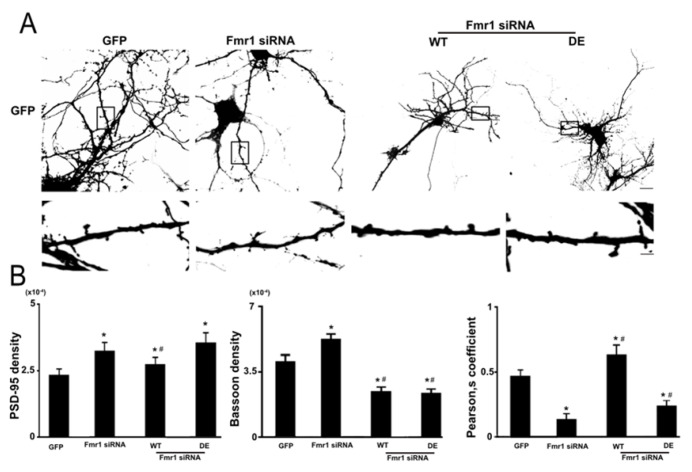
The dendritic spines and synaptic formation defects of mature Fmr1 siRNA neurons are rescued by the RACK1 mutant up-regulation. (**A**) GFP immunofluorescence on 11 DIV neurons co-transfected with GFP and Fmr1 siRNA_/_WT or Fmr1 siRNA/DE. (**Lower**) In the box is the magnification of neuritis. Scale 20μm upper and 5μm lower. (**B**) Graphic of PSD-95, Bassoon density and Pearson’s coefficient of puncta between PSD-95 and Bassoon measured by immunofluorescence experiments as also reported in Figure 1. Graph reporting the correlation measured by Pearson coefficient of the puncta signal between PSD-95 and Bassoon in Supplemental Figure S2. All bar represent mean ± SD; n.70 puncta from three independent experiments. One-way ANOVA with Tukey’s Post Hoc test. * *p* ≤ 0.005, vs. GFP, # *p* ≤ 0.001 vs. Fmr1 siRNA.

Abnormal dendritic development is at the origin of neurodevelopmental disorders such as autism and Fragile X mental retardation syndrome (FXS), a genetic form of autism caused by the loss of translational repressor FMRP [33]. Neurons from FXS mouse models and of human FXS exhibit numerous immature dendritic spines and a translational up-regulation of PSD-95 mRNA, due to loss of its translational repressor FMRP [29]. Considering these findings, we thought that ribosomal RACK1 and FMRP may collaborate in the development of dendritic spines and the translation of *PSD-95* mRNA. To test this hypothesis, we compared the dendritic spine density of the Fmr1 siRNA and up-regulating RACK1_DE-his-myc_ or RACK1_WT-his-myc_ neurons. As previously done, 15 DIV neurons were co-transfected with Fmr1 siRNA, to downregulate the FMRP protein (Supplemental Figure S2B), and with GFP. As expected, Fmr1 siRNA neurons displayed an increase in PSD-95 density as compared to controls (compare Figure 2A,B to GFP in Figure 1D). Moreover, the level of dendritic spine density observed for Fmr1 siRNA neurons was higher than that induced by RACK1_WT-his-myc_ but, interestingly, similar to that displayed by the neurons expressing RACK1_DE-his-myc_. (Figure 2A,B). When RACK1_DE-his-myc_ or RACK1_WT-his-myc_ were co-expressed in Fmr1 siRNA neurons, the PSD-95 density reached values similar to those shown by RACK1_DE-his-myc_ or RACK1_WT-his-myc_ neurons (compare Figure 1D with Figure 2A,B), nullifying the effects of FMRP downregulation. According to these results, it appears the up-regulation of both forms of RACK1 overtakes the effects of the FMRP loss. Thus, it is plausible to hypothesize that ribosomal RACK1 may act downstream of FMRP activity.

Since it has been reported that immature dendritic spines determined by FMRP loss are unable to form synapses [29], we investigated whether the RACK1 up-regulation could normalize this phenotype. To this purpose, as previously performed we used the Pearson’s coefficient to value the number of dots where PSD-95 and the pre-synaptic marker Bassoon co-localized. As expected, the FMRP loss in neurons reduced the PSD-95/Bassoon colocalization with respect to controls, despite they showed an elevated Bassoon and PSD-95 density (compare 2B with GFP in Figure 1D and Supplemental Figure S2A). When we analyzed the RACK1_WT-his-myc_ overexpression in neurons treated with *Fmr1* siRNA, we found an increase in PSD-95/Bassoon colocalization levels similar to those reported in RACK1_WT-his-myc_ neurons and more elevated compared to the rest of the groups (compare Figure 1D with 2B and Supplemental Figure S2). Also, the overexpression of RACK1 mutant resulted in an increase of PSD-95/Bassoon co-localization in neurons with knockdown of *Fmr1*, as compared to *Fmr1* siRNA neurons, but it was less than RACK1_WT-his-myc_ and control neurons (Figure 2B and Supplemental Figure S2).). Interestingly, as observed in the dendritic spine density, the differences of PSD-95/Bassoon colocalization between *Fmr1* siRNA neurons overexpression RACK1_DE-his-myc_ and RACK1_WT-his-myc_ were independent of the Bassoon density, which was similar between them but less than controls (Figure 2B and Supplemental Figure S2). These results indicate that the ribosomal RACK1 may rescue the phenotype of *Fmr1* siRNA neurons and modulate FMRP-mediated *PSD-95* mRNA translation.

### 2.3. Defects of Axon and Dendritic Developments in Fmr1 SiRNA Neurons can Be Rescued by Ribosomal RACK1

**Figure 3:**
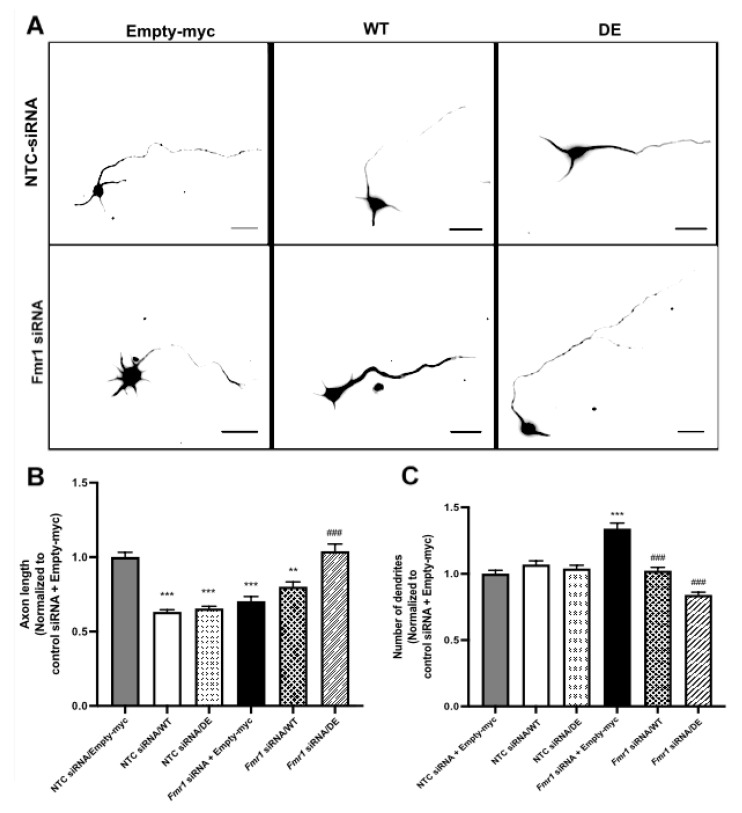
The RACK1 up-regulation rescues the defect induced by Fmr1 siRNA in 2DIV neurons. Overexpression of RACK1 can rescue phenotypic defects underlying Fragile X syndrome. (**A**) E17 mouse cortical neurons were transfected as in Figure 2. Using immunofluorescence (IF), neurons were stained for beta-tubulin and myc, and only myc-stained neurons were analyzed. Fmr1 knockdown results in a reduction in axon length, which is normalized by overexpression of RACK1_DE-his-myc_ (DE in figure). Fmr1 knockdown also increases the number of dendrites which are normalized by overexpression of RACK1_DE-his-myc_. Scale bars, 25 μm. Brightness and contrast were adjusted to optimize the visibility of axons. (**B**) Fmr1 knockdown results in significantly shorter axons. Overexpression of RACK1_DE-his-myc_ normalizes this phenotype. Data normalized to non-targeting control (NTC) siRNA/empty-myc. One-way ANOVA with Tukey’s Post Hoc test, n = 90 for each experimental group. *** *p* < 0.0001, significant as compared to NTC siRNA/Empty-myc, ** *p* < 0.001, significant as compared to NTC siRNA/Empty-myc, ^###^ *p* < 0.0001, significant as compared to *Fmr1* siRNA/Empty-myc. *Fmr1* siRNA + Empty-myc not significantly different from *Fmr1* siRNA/RACK1_WT-his-myc_ (WT in figure), *p* = 0.2488; NTC siRNA/Empty-myc not significantly different from *Fmr1* siRNA + RACK1-DE, *p* = 0.9567. (**C**) Fmr1 knockdown results in a significant increase in the number of dendrites. Overexpression of WT normalizes this phenotype. Kruskal Wallis ANOVA with Dunn’s Post Hoc Test, n = 90 for each experimental group. *** *p* < 0.0001, significant as compared to NTC siRNA/Empty-myc, ^###^ *p* < 0.0001, significant as compared to *Fmr1* siRNA/Empty-myc. NTC siRNA/Empty-myc not significantly different from *Fmr1* siRNA/WT, *p* = 0.9999.

To further support the just-described results, we studied the effects of RACK1 overexpression on axon and dendrite development in mouse cortical neurons transfected with *Fmr1* siRNA and cultured for 2 DIV. Previous studies have reported that mouse hippocampal neurons at this stage have a loss of axon guidance after transfection with *Fmr1* siRNA [32]. Furthermore, neurite outgrowth of iPSC-derived forebrain neurons from individuals with FXS is decreased [34]. Thus, we measured the length of axons and dendrites at 2 DIV of neurons transfected with Fmr1 siRNA and RACK1_DE-his-myc_ or RACK1_WT-his-myc_. In line with our previous results [12], both RACK1_DE-his-myc_ and RACK1_WT-his-myc_ overexpression resulted in a decrease in axon length when compared to control, while the dendrites appeared unaltered at this very early stage (Figure 3A). These results suggest that RACK1 regulates axon length, and its ribosomal function is excluded from this activity. *Fmr1* knockdown significantly reduced axon length at 2 DIV (Figure 3A), in agreement with previous reports [33], and following overexpression of RACK1_WT-his-myc,_ a small increase in axon length resulted in *Fmr1* knockdown neurons, but this was not significant (Figure 3A). However, the axon phenotype in Fmr1 siRNA could be rescued by overexpression of the RACK1 mutant.

Then, we examined the effects of RACK1 overexpression on dendritic spine length in FMRP1 knockdown neurons at 2DIV. In line with previous studies looking at this early developmental stage [35,36,37], we studied whether the initiation of dendrites was affected by *Fmr1* knockdown. We found that *Fmr1* knockdown significantly increased the number of dendrites, and this phenotype was rescued by overexpression of RACK1_WT-his-myc_ (Figure 3B). *Fmr1* knockdown in combination with overexpression of RACK1_DE-his-myc_ also reduced the number of dendrites, but to levels that were significantly less than control. Therefore, the morphological defects shown by *Fmr1* depleted neurons were also rescued by the activity of ribosomal RACK1 in the early stage of neuronal maturation.

### 2.4. RACK1 Regulates the Localization of FMRP on Translational Machinery

**Figure 4:**
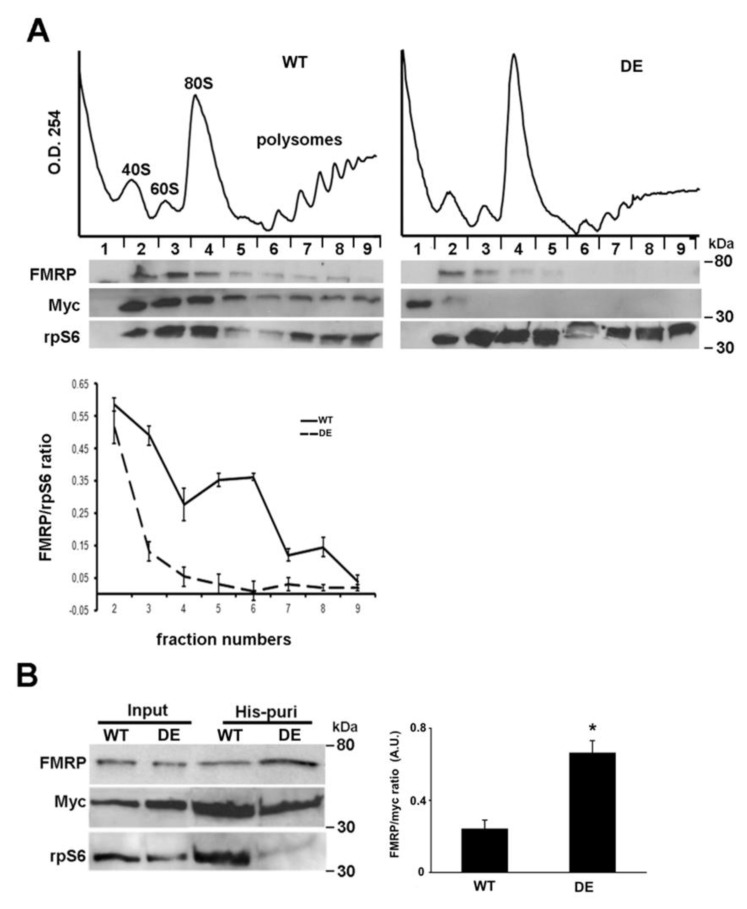
Ribosomal RACK1 recruits FMRP on translational machinery. RACK1 recruits FMRP on polyribosomes. (**A**), Amount of FMRP and rps6, examined by immunoblotting, on ribosomal fractions collected by polysome profiling conducted on SH-SY5Y cells overexpressing Myc-RACK1WT (WT) or Myc-RACK1DE (DE) proteins. (**Lower**) The graphic quantifies the amount of FMRP normalized to the level of rpS6 on ribosome and polyribosome fractions (from 2 to 8 fractions) measured by densitometry on bands related to immunoblottings of three independent experiments. All bar graphs represent the mean and S.D. (**B**) (**Left**) immunoblotting for FMRP, rpS6 and Myc antibodies on proteins eluted by histidine purification from SH-SY5Y cells overexpressing Myc-RACK1WT (WT) and Myc-RACK1DE (DE) proteins. The amount of FMRP purified from eluted imidazole RDE was more elevated than that purified with RWT, while the level of rpS6 was reduced in purified RDE. (**Right**), quantification of FMRP co-purified with WT or DE measured by densitometry on bands related to immunoblottings of three independent experiments. All bar graphs represent the mean and S.D. of FMRP level normalized to the amount of purified Myc-fusion proteins. Student’s T-test was used to calculate *p* values * < 0.01.

Based on these results, we hypothesized that RACK1 might act downstream of FMRP. The observation that RACK1 acts as a ribosomal scaffold on translational machinery for several other RNA-binding proteins [17,38], suggests, reasonably, that this could also occur for FMRP. To verify this hypothesis, we compared the FMRP localization on translational machinery between human SH-SY5Y neuroblastoma cells expressing RACK1_DE-his-myc_ with those expressing RACK1_WT-his-myc_. Immunoblotting for FMRP on translational machinery purified by polysomal profiling revealed that FMRP co-sedimented with RACK1_WT-his-myc_ in fractions containing free ribosomes and polyribosomes, while it was significantly diminished in the polyribosomal fractions of RACK1_DE-his-myc_ cells, where RACK1_DE-his-myc_ was also absent, as previously demonstrated [24] (Figure 4A). This result indicated that the expression of the RACK1 mutant reduced the presence of FMRP on translational machinery. To exclude that this decrease could depend on the different ribosomal amounts between the two cell lines, we performed immunoblotting for ribosomal protein S6 (rpS6) on the same collected fraction. We found that the rpS6 level was similar between ribosomes and polyribosomes of RACK1_DE-his-myc_ and RACK1_WT-his-myc_ cells, and this excluded the possibility of different ribosomal amounts. Consequently, the FMRP decrease did depend on the binding of FMRP to the RACK1 mutant, which could remove the FMRP from translational machinery. To verify this, we tested the interaction of FMRP to mutated and wild type RACK1 by histidine co-purification assay in RACK1_DE-his-myc_ and RACK1_WT-his-myc_ cells. After elution by imidazole, myc immunoblotting on purified proteins confirmed that both RACK1_DE-his-myc_ and RACK1_WT-his-myc_ were similarly purified, while FMRP appeared more co-purified by RACK1_DE-his-myc_ than by RACK1_WT-his-myc_ (Figure 4B). The immunoblotting for rpS6 showed that rpS6 was co-purified by RACK1_WT-his-myc_ purification but not by RACK1_DE-his-myc,_ confirming the reduced affinity of RACK1 mutant with the translational machinery. Altogether, these results indicate that FMRP and RACK1 mutant can form a complex, and FMRP can be removed from translational machinery by binding with RACK1 mutant.

### 2.5. RACK1 and FMRP Are Part of the Same Complex

**Figure 5:**
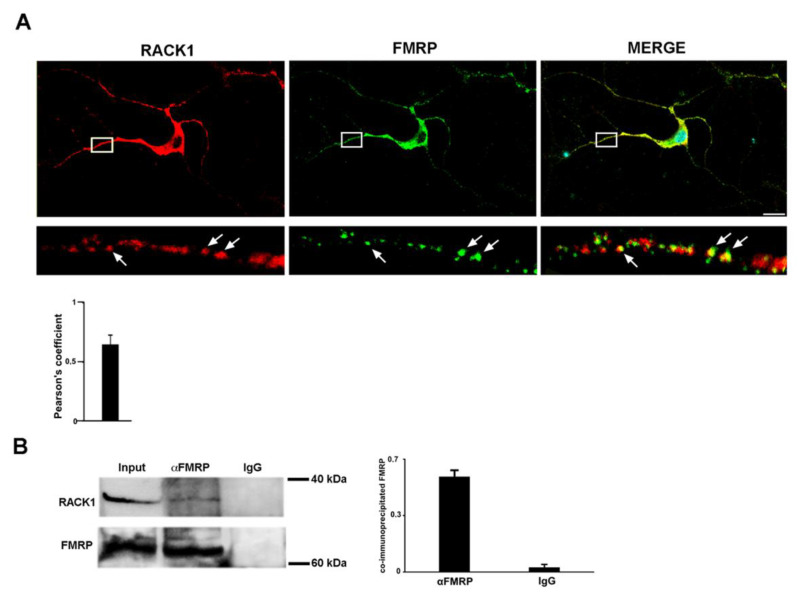
RACK1 and FMRP are part of the same complex. (**A**) RACK1 (red) and FMRP (green) in immunofluorescence experiments co-localize in the soma and partially along neurites of mouse embryonic hippocampal neurons. Inbox, the magnification of one neurite; the arrow indicates the granules where there is colocalization between RACK1 and FMRP. Scale bar: 10μm. Bar grapgh reproteing the Pearson’s coefficient of puncta along between RACK1 and FMRP measured by immunofluorescence experiments. The bar represents mean ± SD; n.70 puncta from three independent experiments. (**B**) Western blotting on eluted protein by RACK1-FMRP co-immunoprecipitation assay from mouse brain. Rabbit IgG was used as a negative control of co-immunoprecipitation. The images are representative of three independent experiments.

To further support previous results, we next determined whether FMRP and RACK1 are found in the same complex. To explore this possibility, we first investigated the localization of RACK1 and FMRP in mouse embryonic hippocampal neurons at 15 DIV. Immunofluorescence experiments revealed that RACK1 and FMRP resided in the soma of the neurons and, as a granular pattern, along axons and dendrites, as previously reported [23,39]. Furthermore, the confocal analysis found that most RACK1 granules coincided with FMRP along the dendrites, and the correspondence was even higher in the soma (Figure 5A). This same result was also obtained in the cytoplasm of SH-SY5Y human neuroblastoma cells (Supplemental Figure S3A). The RACK1-FMRP co-localization induced us to verify t that there was also a biochemical interaction between them. For this, we conducted a co-immunprecipitation assay on total protein extracted from mouse brains using an anti-FMRP antibody to purify FMRP and then verified the RACK1 co-purification by RACK1 immunoblotting. FMRP appeared specifically purified by FMRP immunoprecipitation alone and not by the IgG antibodies used as a negative control, as demonstrated by FMRP immunoblotting. RACK1 was also co-purified by FMRP immunoprecipitation but not in the negative control (Figure 5B), indicating that RACK1 biochemically interacted with FMRP. As before seen for neurons, also in this case RACK1 was specifically co-immunoprecipitated with FMRP in SH-SY5Y cells (Supplemental Figure S3B), furthermore supporting the results observed in vivo. Thus, from these observations, it results that RACK1 and FMRP are part of the same complex in both in vivo and in vitro models.

### 2.6. RACK1 Up-Regulation Decreases FMRP Phosphorylation

**Figure 6:**
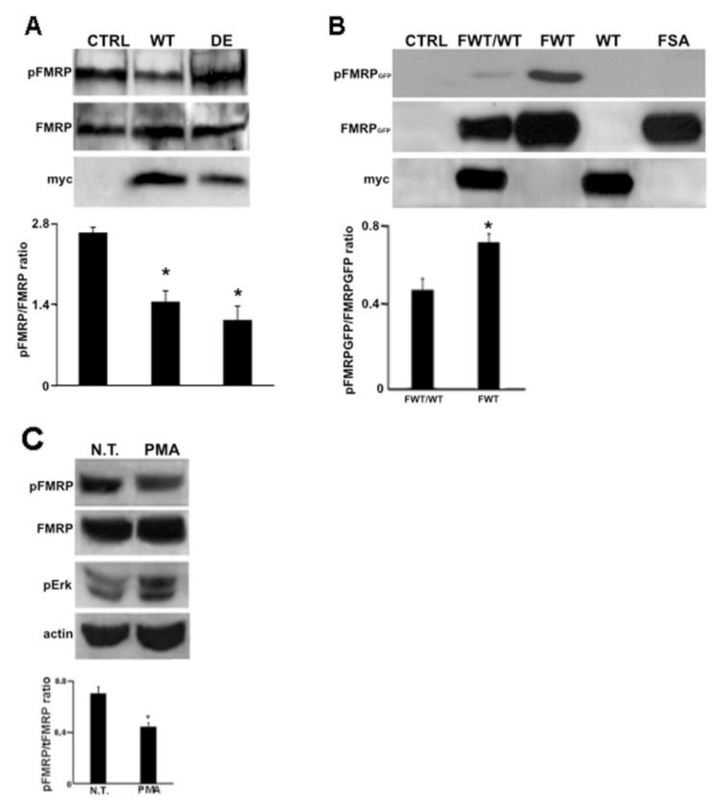
RACK1 up-regulation and PKC activation induce FMRP dephosphorylation at S499. (**A**) Western blotting for phosphorylated FMRP (pFMRP) and total FMRP (FMRP) in control and Myc-RACK1WT (WT) and Myc-RACK1DE (DE) cells. The bar graph summarizes the mean of the pFMRP level normalized to that of FMRP measured by densitometry on immunoblottings of three independent experiments. Student’s T-test was used to calculate *p* values * < 0.01. (**B**) The level of phosphorylated FMRP-GFP in control cells and in cells overexpressing FMRPWT-GFP, FMRPWT-GFP/RACK1_WT-his-myc_ (FWT/WT), RACK1_WT-his-myc_ (WT) and FMRPSA-GFP (FSA). As expected, the S499A mutation in FMRPWT abrogates its phosphorylation. The bar graph summarizes the mean GFP-FMRP level normalized to that of GFP-FMRP measured by densitometry on immunoblottings of three independent experiments. Student’s T-test was used to calculate *p* values * < 0.01. (**C**) Western blotting for phosphorylated FMRP (pFMRP) and total FMRP (FMRP) in PMA treated cells. The immunoblotting for ERK phosphorylation indicated the effect of PMA. The bar graph summarizes the mean of the pFMRP level normalized to that of tFMRP measured by densitometry on immunoblottings of three independent experiments. Student’s T-test was used to calculate *p* values * < 0.01.

The decrease of FMRP on ribosomes may explain the translational control of PSD-95 mRNA found in RACK1 mutant cells. However, we also observed a slight increase in PSD-95 mRNA translation in RACK1_WT-his-myc._ In an attempt to clear this result, we addressed the phosphorylation status of FMRP, which is reported to regulate the translation of PSD-95 mRNA by complexing with miR-125a and Ago2. FMRP is primarily phosphorylated at the conserved site serine 499 and its dephosphorylation by PP2A promotes the translational up-regulation of PSD-95 mRNA. Therefore, we measured the phosphorylated status of this site in SH-SY5Y cells up-regulating RACK1_DE-his-myc_ and RACK1_WT-his-myc_. By immunoblotting, we observed that S499 phosphorylation was decreased both in RACK1_DE-his-myc_ and RACK1_WT-his-myc_ expressing cells when compared to controls (Figure 6A) while the level of FMRP protein was unaltered in all studied cells. This data indicated that the RACK1 up-regulation decreases the S449 phosphorylation of FMRP independently of its binding to ribosomes. To further investigate the FMRP dephosphorylation that occurs as a result of RACK1 up-regulation, S499 phosphorylation was analyzed in cells co-expressing RACK1_WT-his-myc_ with the wild type form of FMRP which was fused to GFP (GFP-FMRP_WT_). Moreover, we overexpressed in SH-SY5Y cells an FMRP mutant, in which serine 499 was substituted with alanine (GFP-FMRP_SA_) to mimic the dephosphorylation and to test the antibody specificity. By immunoblotting, we observed a band in cells expressing GFP-FMRP_WT_, which was reduced when GFP-FMRP_WT_ was co-expressed together with RACK1_WT-his-myc_ and disappeared in GFP-FMRP_SA_ expressing cells (Figure 6B). These results indicated that the band corresponded to phosphorylated FMRP and that RACK1 up-regulation promoted the FMRP dephosphorylation.

Since PKCβII is the main kinase associated with RACK1 [25] and, moreover, may also mediate the mGluR-dependent dendritic localization of FMRP [40], we also wondered whether the PKC activation was involved in the status of FMRP phosphorylation. To stimulate PKC activity, we administrated Phorbol myristate acetate (PMA), a PKC activator, to SH-SY5Y cells and monitored the FMRP phosphorylation by immunoblotting. An increase in extracellular signal-regulated kinases (ERKs) phosphorylation in cells treated with PMA indicated that PKC is active, while in the same cells, PMA also reduced the level of phosphorylated FMRP (Figure 6C). This finding, which is in line with the results obtained from cells overexpressing RACK1, suggests that the activation of PKC activity led to FMRP dephosphorylation.

## 3. Discussion

In this study, we investigated the role of RACK1 during neuronal development in vitro and find that RACK1 up-regulation correlates with the progressive maturation of mouse embryonic hippocampal neurons up to 17 DIV. When the ectopic expressing of a RACK1 mutant exhibits low affinity for ribosomes [41], we found a decrease in dendritic arborization and, by contrast, an increase in dendritic spine density, indicating that ribosomal RACK1 is mainly involved in neuron differentiation. Furthermore, at the molecular level, we find that RACK1 overexpression induces the translation of PSD-95 mRNA. Altogether these results appear to recapitulate those caused by FMRP loss in neurons, which is well known to cause the formation of numerous immature dendritic spines and up-regulation of the PSD-95 mRNA translation [42]. Having these considerations in mind, Thus, we upregulated RACK1 in FMRP knockdown neurons and find that expression of the ribosomal RACK1 mutant rescues the dendritic spine and neurite defects caused by loss of FMRP. In an attempt to molecularly explain these results, we uncovered that the depletion of ribosomal RACK1 from translational machinery also decreases the association of FMRP with ribosomes and, particularly, polyribosomes; these data suggest that RACK1 may recruit FMRP on translational machinery and result in *PSD-95* mRNA translation.

In the past, we and others reported that the RACK1 regulates dendritic arborization and growth cone morphology [12,23]. Similarly, RACK1 controls PC12 cell differentiation in neurons by modulating the FAK activity [43]. The neuron axon growth of *C. elegans* neurons is regulated by the interaction of RACK-1 with UNC-115/abLIM, an actin-binding protein required for the actin cytoskeleton [44]. Also, severe defects in cerebellar morphogenesis in the mouse model are caused by the selective loss of RACK1 in neuronal stem cells or granule cell progenitors [45]. Finally, our study found that the ribosomal RACK1 is involved in neuronal development. Taken together these findings seem to indicate that different forms of RACK1 may promote neuronal development. However, it is also plausible to hypothesize that there is a link among these different forms of RACK1. Analysis of the crystallographic structure of the RACK1-40S complex may clear this discrepancy. In this structure, although most of RACK1 is covered when bound with the 40S ribosome, RACK1 still exhibits several binding sites for other molecules such as FAK, Integrin and Src and, also, for eIFs [46]. This suggests that the ribosomal RACK1 may promote the translation of specific mRNAs after the binding of these molecules. This possibility is also supported by the presence of RACK1, 18S rRNA, FAK and integrins in the spreading initiation centers (SIC), which are essential structures for cell adhesion and migration. The activation of the adhesion pathway in SICs may stimulate the interaction of FAK and/or integrins with ribosomal RACK1, leading to a localized translation of specific mRNAs to produce fundamental proteins needed for cell adhesion and motility [47,48]. Thus, this model may also apply to neuronal development where the binding of specific RACK1 partners to ribosomal RACK1 may stimulate the translation of specific mRNAs for neuron differentiation.

Our study finds that the up-regulation of ribosomal RACK1 also alters the number of dendritic spines, which are the postsynaptic sites of many synapses [49]. In contrast to dendritic arborization decrease, neurons expressing the RACK1 mutant show a consistent increase in dendritic spines, which is also confirmed by an increase in PSD-95 density and corresponds to the up-regulation in *PSD-95* mRNA translation. Elevated levels of PSD-95 and an increase in spine density are reported in neurodevelopment disorders such as autism and Fragile X syndrome. In our study, up-regulation of the RACK1 mutant in neurons with FMRP knockdown results in a PSD-95 density that is at a level similar to that of neurons expressing the RACK1 mutant alone; thus, this result seems to exclude FMRP loss for the *PSD-95* mRNA translation. To explain this data, we speculate that ribosomal RACK1 may act downstream of FMRP activity. One hypothetical mechanism for this hypothesis is that ribosomal RACK1 may act as a scaffold for FMRP on ribosomes. This hypothesis is supported by our results where the binding of FMRP to the RACK1 mutant in SH-SY5Y neuroblastoma cells decreases the level of FMRP on ribosomes and polyribosomes. Furthermore, it is supported by cryoelectron microscopic reconstruction of the 80S-FMRP complex displaying a direct binding of FMRP to ribosomes [38]. It is important to note that Chen et al. report that FMRP binds the L5 ribosomal protein on the 80S subunit, thus we cannot exclude an indirect effect of RACK1 mutant on the binding of FMRP to ribosomes. Indeed, the ribosomal RACK1 mutant may remove from the translational machinery other FMRP ribosomal interactions such as the Ago2-miR125a complex; the formation of this complex is promoted by FMRP phosphorylation to inhibit the PSD-95 mRNA translation [42]. In this context, it is also interesting that ribosomal RACK1 recruits the RISC complex on translational machinery in humans and *C. Elegans* [50]. However, further experiments are required to demonstrate that ribosomal RACK1 is the main ribosomal partner of FMRP.

As previously reported, FMRP phosphorylation is required to repress the translation of PSD-95 mRNA [42]. In our study, we show that the RACK1 wild type up-regulation stimulates PSD-95 mRNA translation, albeit less than the RACK1 mutant. We also observe a decrease in FMRP phosphorylation both in RACK1_DE-his-myc_ and RACK1_WT-his-myc_ cells, indicating that the binding of RACK1 to the ribosome is not a prerequisite for the FMRP dephosphorylation. Furthermore, when SH-SY5Y cells are stimulated by PKCβII kinase, one of the kinases binding RACK1, FMRP phosphorylation is decreased. PP2A is the main phosphatase dephosphorylating FMRP at S499 [51] and its dephosphorylation inhibits the formation of the Ago2-miR125a complex so that PSD-95 mRNA can be translated after mGluR signaling activation [42]. These findings suggest that the RACK1 up-regulation or the PKCβII activation may stimulate the PP2A activity to dephosphorylate FMRP. This speculation is supported by the finding that RACK1 modulates the PP2A phosphatase in MCF-7 cells [52] and that PKC inhibition impairs the mGluR-induced dendritic localization of FMRP [40]. However, further experiments will be required to demonstrate this hypothesis and to verify that the formation of the Ago2-miR125a complex on translation machinery is influenced by RACK1 up-regulation or PKCβII activation. Moreover, it must be investigated whether FMRP dephosphorylation under these conditions influences the association of FMRP to ribosomes or polyribosomes, as such reported by Ceman et al. [53]

## 4. Materials and Methods

### 4.1. Primary Neuronal Culture and Transfection

Embryonic hippocampal tissues (E17,5) were used for neuronal primary culture. After dissociation, trypsin digestion for 15 min was performed, and the cell dissociated for a plate at 5 × 10^4^ on poly-lysin glass. The cells were grown in a neurobasal medium supplemented with B27, antibiotic, 2mM glutamine and glutamate. The neurons were cultured for immunofluorescence for the analysis of dendritic spines at DIV 15. At DIV 11 neurons were transfected with GFP and plasmid shFMRP, RACK1_WT-his-myc,_ RACK1_DE-his-myc_ and RACK1 siRNA plasmids in ratio 3:1 (GFP:other plasmids) using Lipofectamine 2000 according to the manufacturer protocol. With this ratio, every GFP positive neuron also contains the other plasmid After three days of transfection, neurons were fixed with PFA4% and sucrose 4% and used for dendritic spine analysis. For 2DIV experiments, the day of the plug was considered as embryonic day 0 (E0) and cortices were dissected from E17 embryos of either sex. Dissociated cortical neurons were transfected with both a RACK1 construct or GFP or *Fmr1* siRNA. Transfections were performed using an Amaxa Nucleofector 4D system (program CU-133 and Amaxa P3 Primary Cell 4D Nucleofector Kit). Neurons were then plated in Neurobasal with Glutamax and B-27 (all Life Technologies) on acid-rinsed coverslips (Carolina Biological) pre-treated with 30 μg/mL poly-L-lysine (Sigma-Aldrich). After cells adhered to the coverslips (approximately 2 h), the media was changed to fresh Neurobasal with Glutamax and B-27, and cells were cultured for 2 DIV at 37 °C in 5% CO_2_. Neurons transfected were used to measure the axon length and dendritic number.

### 4.2. Dendritic Spines Analysis

Neurons transfected with GFP and shFMRP, RACK1_WT-his-myc_ and RACK1_DE-his-myc_ were permeabilized and non-specific binding sites of proteins blocked with Goat Serum Dilution Buffer (GSDB; 15% goat serum, 0.3% Triton X-100, 450 mM NaCl, 20 mM phosphate buffer, pH 7.4) for 30 min. The following primary antibodies were used: cicken anti-Bassoon (Synaptic Systems, 1:400), mouse anti-PSD95 (UC Davis/NIH NeuroMab Facility, CA, 1:100) and mouse anit-Myc (9B11, Cell Signalling, 1:1000). Alexa fluorescent secondary specific antibodies were used for immunolabeling and DAPI staining was performed. Co-labelling GFP and myc immunofluorescences were used to monitor that every GFP positive neurons also expressed the myc-constucts. The operator-blind to the sample treatment-measured the number of Bassoon pre-and PSD-95 postsynaptic positive juxtaposed puncta formed on one selected segment of the proximal dendrite of GFP neurons positive for each image. The number of dendrites represents the number of neurons analyzed in at least 3 to 4 independent experiments. Twenty neurons at least for each independent experiment and condition and counted using Neurolucida software. A total of 320 dendrites over a length of 50 μm starting 20 μm from the nucleus was analyzed. The average of all analyzed neurons in each independent experiment was considered for the statistics. Quantification was performed with GraphPad Prism. Images were acquired using a Leica SP8I confocal microscope equipped with an ACS APO 40x or 63x oil immersion objective.

The Scholl’s analysis to measure the dendritic arborization was performed as in Ceci et al. [24].

### 4.3. Axon Length and Dendritic Number Analysis

IF images were visualized using a Nikon Ti-2E microscope at a magnification of 20X (0.45 N.A.). Images were captured with a Hamamatsu sCMOS camera (ORCA Flash4.0 V3), using NIS Elements software. Axon length was quantified using Fiji software (Schindelin et al., 2012). We can reliably define the axon as the longest neurite, which extends at least three times the length of the next longest neurite. Axon length was determined by measuring the length of the primary axon (longest neurite) from the cell body to the center of the axonal growth cone; it does not include the length of any branches coming off the primary axon. The number of dendrites were counted manually. Only those neurons that stained for the myc tag were included in the analysis. Cells were fixed with 4% paraformaldehyde in phosphate buffered saline. If experiments were performed as previously described [54]. and the following primary antibodies were used: mouse anti-beta tubulin (E7 clone) antibody (1:1000; DSHB) and rabbit anti-myc antibody (1:500; Abcam). The following secondary antibodies were used: goat anti-mouse Alexa 488 (Life Technologies) and donkey anti-rabbit Cy3 (Jackson ImmunoResearch).

### 4.4. Cell Culture, Transfections and Treatments

Human neuroblastoma SH-SY5Y cells, obtained from American Type Culture Collection (ATCC, Rockville, MD), and stable SH-SY5Y clone cells for Myc-RACK1_WT_, GFP-FMRP_WT_ and GFP-FMRP_S499A_, produced as previously reported in [24], were cultured in DMEM/F12 medium containing 10% fetal bovine serum (FBS) and antibiotics (50 U/mL penicillin and 50 µg/mL streptomycin) at 37 °C in 5% CO_2_/95% air. Embryonic primary cultures were produced and cultured as reported in [24]. A stable Myc-RACK1_WT_ cell line was transiently transfected with GFP-FMRP_WT_ or GFP-FMRP_S499A_ cDNA using the manufacturer’s protocol of Lipofectamine 2000 (Invitrogen).

For pharmacological treatments, wild type and transfected SH-SY5Y cells were starved 24 h and then treated with 50 nM Phorbol 12-Myristate 13-Acetate (PMA, Thermofisher) for 6 h at 37 °C.

### 4.5. Immunofluorescences and Antibodies

Immunofluorescence staining on neurons and SH-SY5Y cells was performed as previously described [24]. Briefly, after two rinses with Phosphate Saline Buffer (Na_2_HPO_4_ 10mM, KH_2_PO_4_ 1.8mM, NaCl 137mM, KCl 2.7mM, pH 7.4), SH-SY5Y and embryonic primary cells were fixed with 4% paraformaldehyde (PFA) for 10 min at room temperature, then rinsed again with PBS and permeabilized with PBS-Triton-X 0.5% for 10 min at room temperature. After blocking in PBS-BSA 2% at 37 °C for 30 min, cells were incubated for 30 min at 37 °C with the rabbit monoclonal anti-FMRP (Abcam, 1:100) and mouse monoclonal anti-RACK1 (BD Biosciences, 1:200) primary antibodies (dissolved in blocking solution). After washes with PBS, the following secondary antibodies were incubated for 30 min at 37 °C in the dark: goat anti-mouse, goat anti-rabbit (Alexa Fluor^®^ secondary antibodies, Molecular Probes), dissolved 1:500 in PBS-DAPI. After mounting with 80% glycerol, cells were visualized by fluorescence microscopy (Axio Scope, Zeiss, Viterbo, Italy).

### 4.6. Polysomal Profile

The polysomal profiling was led as previously seen [24]. Briefly, growing cells were lysed in polysomal buffer (10mM Tris–HCl,150mM NaCl, 10mM MgCl2 and 0.1% TritonX-100). MgCl2 was substituted with 5 mM EDTA for polysome profiles in the presence of EDTA. The total lysate was clarified by centrifugation at 15,000× *g* for 5 min at 4 °C and the supernatant was loaded on a continuous sucrose gradient of 15–50% in 10mM Tris–HCl, 150mM NaCl, 10mM MgCl2 or 10 mM EDTA. After ultracentrifugation at 4 °C in an SW41Ti Beckman rotor at 37,000 rpm for 2 h, absorbance at 254 nm was recorded by BioLogic LP software (BioRad) and fractions (1 mL each) were collected. The proteins in the collected fractions were precipitated incubating them in 10% trichloroacetic acid (TCA) for 30 min in ice and then centrifuged at 15,000× *g* for 15 min. The resulting protein pellets were then resuspended for subsequent analysis.

### 4.7. Histidine Pull-Down

Transfected cells were lysed in lysis buffer (10 mM Tris-HCl, 100 mM NaCl, 10 mM MgCl_2_ and 0.1% Triton C-100) and the total lysate was clarified by centrifugation at 14,000× *g* for 5 min at 4 °C. Then, protein extracts were incubated for 1 h with nickel affinity resin (BioRad), pre-equilibrated with lysis buffer. The resin was then extensively washed with 20 mM Imidazole dissolved in lysis buffer and finally eluted with 300 mM Imidazole in lysis buffer. The eluted proteins were then precipitated with 10% trichloroacetic acid (TCA) for 30 min in ice, and then centrifuged at 14,000× *g* for 15 min. The resulting protein pellets were then resuspended in Tris-HCl 1M for subsequent analysis.

### 4.8. Co-Immunoprecipitation Assay

For tissue co-immunoprecipitation, mouse brains were collected and mechanically homogenized in lysis buffer (10 mM Tris-HCl, 100 mM NaCl, 10 mM MgCl_2_ and 0.1% Triton C-100). The whole-brain lysate was then centrifuged for 20 min at 12,000× *g* at 4 °C and the supernatant was incubated overnight with the anti-FMRP (Abcam, 1:100) primary antibody. Immunocomplexes were then captured by Protein A-agarose (Sigma-Aldrich) during a 2 h incubation step. Next beads were excessively washed with lysis buffer, and bound proteins were eluted in denaturing Laemmli SDS gel loading buffer assisted by incubation at 95 °C for 5 min. Finally, eluted proteins were processed for immunoblotting SDS-PAGE analysis. For SH-SY5Y cell co-immunoprecipitation, 20 × 106 cells were washed twice in PBS 1X and scraped with lysis buffer as previously reported. Next, co-immunoprecipitation was conducted for brain tissue immunoprecipitation.

### 4.9. Immunoblottings and Antibodies

Immunoblotting was performed on protein extracts obtained with lysis buffer as previously seen for histidine pull-down. Equal amounts of proteins were loaded on a 10% SDS-PAGE and transferred to the PVDF membrane (Millipore). After blocking in 5% Bovine Serum Albumin in PBS1X for 30 min at 37 °C, blots were incubated with the following primary antibodies: mouse monoclonal anti-RACK1 (BD Biosciences, 1:2000), mouse monoclonal anti-β-actin (Sigma, 1:1000), mouse monoclonal anti-puromycin (Millipore, 1:10,000), mouse monoclonal anti-Myc 9B11 (Cell Signaling, 1:1000), rabbit monoclonal anti-FMRP (Abcam, 1:1000), rabbit polyclonal anti-phospho-FMRP (Abcam, 1:500), rabbit monoclonal anti-phospho-ERK (Cell Signaling, 1:1000), rabbit monoclonal anti-phospho-rpS6 (Cell Signaling, 1:1000). Secondary HRP-conjugated anti-mouse or anti-rabbit antibodies and ECL reagent (1:5000, GE Healthcare) were used. For RACK1, mouse HRP-conjugated anti-IgM (1:5000, Sigma) was used.

### 4.10. RNA Isolation and QRT-PCR

Total RNA was purified from immunoprecipitates and ribosomal fractions with TriReagent (Invitrogen) according to the manufacturer’s protocol. The purified RNA was used for qRTPCR. The first strand cDNA template was synthesized from 500 ng of total RNA using random primers and Superscript III reverse transcriptase (Invitrogen, USA). All reactions were performed with SYBR Green PCR Master Mix (BioRad) and carried out in the iCycler (BioRad). Primers for Quantitative PCR (QTR-PCR) analysis were designed with the assistance of Universal Probe Library Software (Roche Applied Science). The Following primers were selected to amplify: Homo sapiens PSD-95, forward GCATGCTGGGAGCTGTAGT and reverse ATCCCTCTAAGTCAGCGGAAC; Homo sapiens RNA, 18S (ribosomal 1 forward) 59-AGGGCAGGGACTTAATCAACGC-39 and reverse 59-GTTGGTGGAGCGATTTGTC TGG-3. Relative change of mRNA amount was calculated based DCt method, as described in [55].

### 4.11. Statistical Analysis

The results are presented as the mean ± standard error of the mean (SEM) or mean ± deviation standard (SD). IBM SPSS Statistics for Windows, version 23.0 (IBM Corp.) and GraphPad Prism software (5.0 version; GraphPad Software, Inc.) were used for the statistical analyses. A two-tailed unpaired Student’s *t*-test and one-way analysis of variance (ANOVA) with Tukey’s post hoc test were used for comparisons between two or multiple groups, respectively. A value of *p* < 0.05 was considered to indicate a statistically significant difference. Error bars represent the standard error of the mean from a minimum of three independent experiments. The Pearson’s coefficient was measured as in Russo et al. [24]

## 5. Conclusions

In conclusion, here we find that ribosomal RACK1 is required for neuronal development and the loss of RACK1 ribosomal function may contribute to neurodevelopment disorders such as autism. As previously reported for ZBP1 and TDP-43, ribosomal RACK1 is important for scaffolding FMRP in developing neurons, and, thus, in this context, may regulate *PSD-95* mRNA translation by FMRP.

## Supplementary Materials

The following supporting information can be downloaded at: https://www.mdpi.com/article/10.3390/ijms231911857/s1.
