# Supplementary material for: Ribosomal RACK1 Regulates the Dendritic Arborization by Repressing FMRP Activity
Source: Int J Mol Sci. 2022 Oct 6;23(19):11857. doi: 10.3390/ijms231911857 (PMC9569565; doi:10.3390/ijms231911857)
Supplement: Supplementary file 1 [file ijms-23-11857-s001.zip › ijms-1839345-supplemental.pdf]

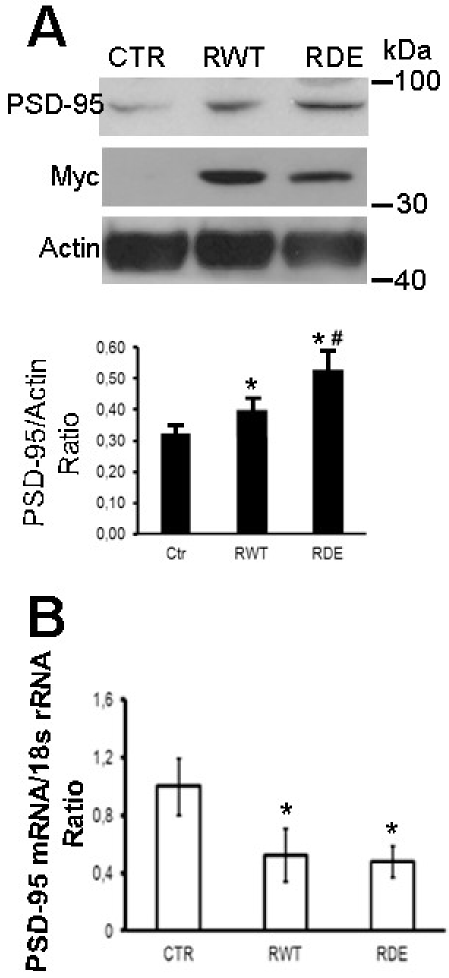

Figure Supplementary S1. RACK1 up-regulation enhances the translation of PSD-95 protein**.** (**A**) western blotting for PSD-95 protein in SH-SY5Y cells overexpressing RWT and RDE and control. The bar graph below quantifies the level of PSD-95 protein normalized to that of actin protein by densitometry on bands related to immunoblottings of three independent experiments. All bar graphs represent the mean and S.D. Student's T-test was used to calculate P values * < 0,01, # $p \leq 0.05$. (**B**) of PSD-95 mRNA. The bar graph shows the level of PSD-95 mRNA, measured by qRT-PCR, in control and RACK1WT or RACK1DE overexpressed cells. The amount of PSD-95 mRNA was normalized to that of rRNA 18S. All bar graphs represent the mean and S.D of three independent experiments. Student's T-test was used to calculate P values * < 0,01.

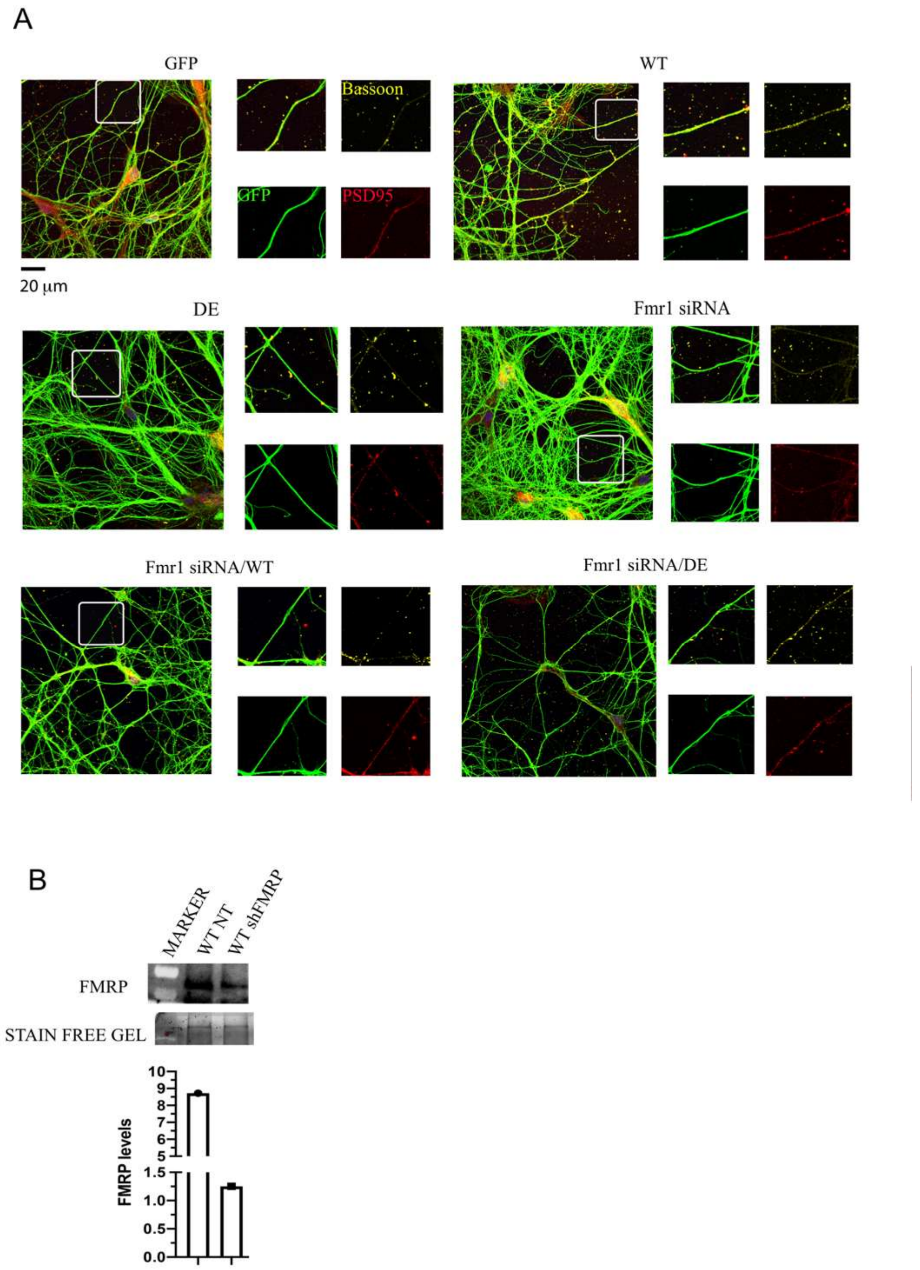

Supplemental Figure S2. (**A**) Immunofluorescence for GFP, PSD-95 and Bassoon performed on 15DIV neurons transfected with all constructs as reported in the text. The positive dots for PSD-95 and Bassoon were used to measure PSD-95 and Basson density and the Pearson's coefficient. (**B**) Immunoblotting for FMRP protein in 15DIV transfected with Fmr1 siRNA. The bar quantifies the level of FMRP protein normalized to ponceau by densitometry of three independent experiments. All bar graphs represent the mean and S.D of three independent experiments

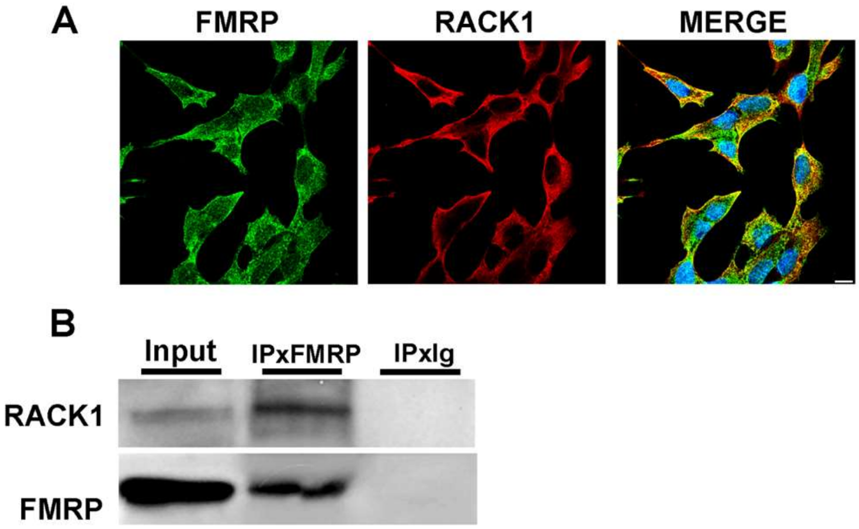

Figure Supplementary S3: (**A**) RACK1 (red) and FMRP (green) in immunofluorescence experiments colocalize in SH-SY5Y cells. Scale bar: 10μm. (**B**) western blotting on eluted protein by RACK1-FMRP coimmunoprecipitation assay. RACK1 co-purified with FMRP but it failed in IgG immunoprecipitation. Rabbit IgG was used as a negative control of co-immunoprecipitation. The images are representative of three independent experiments.
